# Genetic diversity in German Pomeranian Coarsewool sheep and the possibility of breeding for maedi-visna resistance

**DOI:** 10.5194/aab-68-517-2025

**Published:** 2025-08-08

**Authors:** Cassandra Frölich, Martin Ganter, Olusegun Olaniyi Adeniyi, Gesine Lühken

**Affiliations:** 1 Clinic for Swine and Small Ruminants, University of Veterinary Medicine Hannover, 30173 Hanover, Germany; 2 Institute of Animal Breeding and Genetics, Justus Liebig University of Giessen, 35390 Giessen, Germany

## Abstract

Knowledge of genetic diversity among sheep breeds is essential for conserving genetic resources, in particular when selective-breeding programs are taken into consideration, for example, to enhance disease resistance. Maedi-visna virus (MVV) is an incurable, progressive sheep disease caused by a small-ruminant lentivirus (SRLV) infection. In certain sheep breeds, MVV susceptibility has been associated with the allele E of the ovine transmembrane protein gene (*TMEM154*), whereas the allele K in a homozygous state is protective. The present study estimated the genetic diversity of the native German Pomeranian Coarsewool (RPL, “Rauhwolliges Pommersches Landschaf”) sheep breed and evaluated the possibility of breeding for MVV resistance. A total of 185 RPL sheep were genotyped using the Illumina ovine 50K single-nucleotide polymorphism (SNP) BeadChip to calculate genetic diversity indicators such as effective population size (Ne ${}_{13}=$ 116) and Wright's F statistical indices ( $F_{\mathrm{IS}}=$ 0.005). The mean inbreeding coefficient based on runs of homozygosity ( $F_{\mathrm{ROH}}$ ) was 6.2 %, with a moderate correlation (0.42) with the pedigree-based coefficient ( $F_{\mathrm{PED}}$ ). Overall, the calculated genetic diversity parameters indicate that the diversity in the RPL breed is not at risk. Thus, breeding for MVV resistance would be possible without a major loss of genetic diversity due to the relatively high frequency of the allele K (54 %). However, selection for the *TMEM154* allele K across the entire breed is not advised until further studies confirm its protective effect against MVV in the RPL breed.

## 1 Introduction

Sheep (*Ovis aries*) are descendants of the wild Asian mouflon and were one of the first domesticated species around 10 000 years ago (Scherf and Pilling, 2015). Since domestication, 1500 unique sheep breeds have developed worldwide due to local environmental and human management adaptations (Scherf and Pilling, 2015; Yang et al., 2016). These various influences have resulted in great genetic diversity within the sheep population, but this diversity is currently threatened by economic forces and modernized agriculture (Tisdell, 2003). More than any other continent, Europe is home to almost half of all sheep breeds (Rischkowsky and Pilling, 2007). Of these European breeds, 14 % are already extinct, 43 % are at risk of extinction, and only 15 % are considered “safe” (FAO, 2024). The loss of this diversity is also leading to increased inbreeding, resulting in a higher risk of recessive genetic defects and jeopardizing genetic improvement and adaptation to future environmental, food production, and disease challenges (Keller and Waller, 2002; Rege and Gibson, 2003). The preservation of the genetic diversity of local breeds is considered imperative due to the role of this diversity as a genetic resource for potential future benefits and as cultural heritage (Mendelsohn, 2003). In this context, studying the genetic diversity of well-adapted local breeds is important to develop suitable breeding programs that will help to maintain and improve their genetics (Scherf and Pilling, 2015).

**Figure 1:**
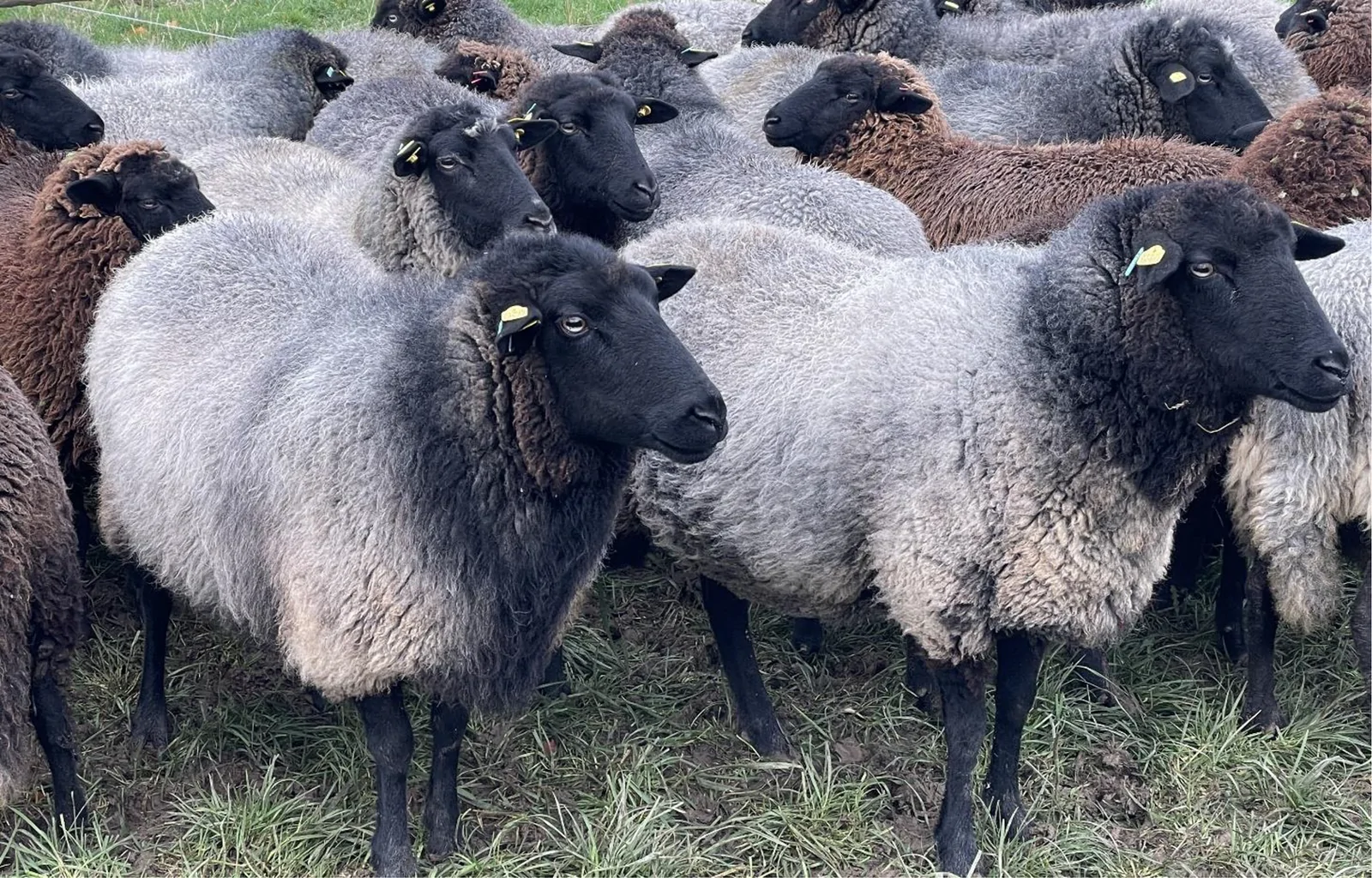
Adult RPL ewes (blue-gray wool) with lambs (brown wool).

In Germany, 20 of the approximately 40 native sheep breeds are endangered, particularly the so-called “land sheep breeds” such as the Pomeranian Coarsewool sheep (RPL, “Rauhwolliges Pommersches Landschaf”) (GEH, 2024; Scherf, 2000). The RPL is a medium-sized, polled sheep with gray to blue-gray wool on their bodies, whereas their heads and legs are black and free of wool (Gaede, 1926; Grumbach, 2002) (Fig. 1). This breed originated from the island of Rügen in Pomerania and resulted from crosses between the extinct German Zaupelschaf and Hannoversche Landschaf breeds (Gaede, 1926; Grumbach and Zupp, 2008). In 1936, the RPL population comprised 70 200 animals. This number increased to 110 000 after the Second World War due to the breed's high robustness and low maintenance requirements (Grumbach, 2002). However, as the economic situation stabilized, sheep breeds with higher productivity were favored, resulting in a decline in the RPL population (Grumbach and Zupp, 1992). In 1962, the last three registered RPL breeding sheep were shown at an exhibition (Grumbach, 2002).

Conservation efforts with respect to this indigenous German breed started in 1982, with 46 ewes, 7 rams, 8 female yearlings, and lambs, as part of a program that was initiated by the United Nations (UN) resolution for the protection of endangered cultural breeds (Grumbach, 2002; Grumbach and Zupp, 1992). Initially, there were seven original Pomeranian ram lines, and these lines were later supplemented by the S-line from a breeder in southwestern Germany (IGRPL, 2024). Due to the limited original population size and the occurrence of bottlenecks, a line-rotation breeding method was implemented to avoid further inbreeding (Grumbach, 2002). This method is still used today, maintaining almost 3500 herdbook-registered RPL sheep in Germany (VDL, 2023). Although the population size is increasing, the RPL breed remains at risk according to the German Society for the Conservation of Old and Endangered Livestock Breeds (GEH). This is because the GEH considers not only the current population but also other factors with an influence on the situation of a breed, e.g., the number of active breeders, offspring rates, and the existence of state subsidy programs (GEH, 2024).

An additional challenge in the conversation of genetic diversity is selective breeding for phenotypic traits or for functional traits, e.g., disease resistance. The RPL breed is known to be less susceptible to certain diseases, such as foot rot and scrapie (Drögemüller et al., 2004; Grumbach and Zupp, 2008; Rischkowsky and Pilling, 2007). For several years, prion protein gene (*PRNP*) alleles associated with scrapie resistance have been considered in the selection of sires (Grumbach, 2002). In the future, breeding for maedi-visna virus (MVV) resistance could be an additional opportunity. In many breeds, the susceptibility to MVV is associated with the ovine transmembrane protein 154 gene (*TMEM154*) (Heaton et al., 2012). At position 35 of the TMEM154 protein, the amino acid lysine (K allele) has a protective effect in certain sheep breeds in the homozygous form, while the presence of the amino acid glutamate (E allele) is associated with susceptibility. In the RPL breed, this association between *TMEM154* genotypes (hereafter always referring to the variation at position 35 of the protein) and MVV seropositivity showed a trend towards significance ( p= 0.09) (Frölich et al., 2024). One discussed reason for the lack of significance was the low number of serologically MVV-positive sheep (30 out of 849), of which 26 RPL sheep carried the *TMEM154* genotype EK/EE and four exhibited the genotype KK. As the putative protective genotype KK and allele K were present in 28 % and 53 % of the genotyped RPL population, respectively, selective breeding could be an additional tool to eradicate MVV in infected flocks or even in the whole breed.

Before considering selective breeding for the *TMEM154* allele K, it is crucial to assess the current genetic diversity of this breed and understand how it might be influenced by selection. Various studies have calculated population genetic parameters such as effective population size (Ne), expected heterozygosity (He), observed heterozygosity (Ho), and Wright's F statistical indices ( $F_{\mathrm{IS}}$ ) to describe the genetic diversity of different sheep breeds (Adeniyi et al., 2022; Al-Mamun et al., 2015; Sveistiene and Tapio, 2021). However, the state-of-the-art method for inbreeding analysis using genotyping data is the calculation of the inbreeding coefficient ( $F_{\mathrm{ROH}}$ ) based on runs of homozygosity (ROH) (Peripolli et al., 2017). Many studies have shown that this coefficient is more accurate than the pedigree-based inbreeding coefficients ( $F_{\mathrm{PED}}$ ) (Burren et al., 2016; McQuillan et al., 2008; Meyermans et al., 2020a; Wiener et al., 2017).

The aim of the present study was to analyze the genetic diversity of the native German RPL breed and to investigate the possibility of selection for the *TMEM154* allele K without compromising genetic diversity by generating and analyzing Illumina ovine 50K single-nucleotide polymorphism (SNP) chip genotyping data of this breed. Various diversity parameters were calculated not only for the entire RPL breed but also for different ram lines, to assess the effect of the registration and consideration of ram lines in breeding decisions, and for *TMEM154* genotypes, to evaluate the potential loss of genetic diversity if sheep with certain genotypes were to be selected in the future.

## 2 Methods and materials

### 2.1 Animal sampling and genotyping

As part of this and a related project (Frölich et al., 2024), veterinarians collected ethylenediaminetetraacetic acid (EDTA) blood samples from 54 RPL flocks on voluntarily participating farms across nine German federal states (Baden-Württemberg, Brandenburg, Hesse, Lower Saxony, Mecklenburg–Western Pomerania, North Rhine-Westphalia, Saxony, Saxony-Anhalt, and Schleswig-Holstein). A representative set of not closely related RPL sheep (no first- or second-degree relatedness) was chosen for genotyping based on pedigree information. Sheep from all eight ram lines were included in the sample set; for details, see Table 1. The number of sampled sheep per flock ranged from one to nine, proportional to the flock size. A total of 192 samples from RPL sheep (187 purebred and 5 crossbred) born between 2013 and 2022 were sent to Neogen (Ayr, Scotland, UK) for genotyping using the OvineSNP50K BeadChip v.3 (Illumina, San Diego, CA, USA).

**Table 1:** Selection of RPL sheep for the SNP chip divided by ram lines, sex, and *TMEM154* genotype results.

Ram line	Sex		*TMEM154*			Total
			genotype			
	Male	Female	EE	EK	KK	
Line 1	3	13	5	8	3	16
Line 2	7	10	8	5	4	17
Line 3	3	16	4	8	7	19
Line 4	9	20	6	12	11	29
Line 5	10	20	5	18	7	30
Line 6	7	10	2	12	3	17
Line 7	10	20	3	18	9	30
Line S	5	6	2	5	4	11
Unknown line	4	19	4	13	6	23
Total	58	134	39	99	54	192

### 2.2 Genome-wide SNP and *TMEM154* genotyping

All 192 samples were genotyped with the OvineSNP50K BeadChip. OvineSNP50k BeadChip results for SNP OAR17_5388531 were checked for the nucleotide substitution G > A in the coding region of *TMEM154*, resulting in the amino acids glutamate (E) or lysine (K) at position 35 in the protein. Except for one, the samples were additionally genotyped for the *TMEM154* genotypes using KASP technology (LGC, Hoddesdon, UK), as previously described by Frölich et al. (2024) and Molaee et al. (2018), to check the reliability of the SNP chip data.

### 2.3 Quality control analysis

A total of 192 animals and 61 918 SNPs were available for analysis. Prior to quality control (QC) analysis of the data, the “–related” function in KING version 2.2.4 was used to check the relatedness of the animals (Chen, 2024). One of each related animal of pairs with an identify-by-descent (IBD) value > 0.177 (second-degree relationship) was removed, resulting in the exclusion of three animals. After that, QC was performed using PLINK version 1.9 (Chang et al., 2015). SNPs genotyped in less than 95 % of the animals or with a minor allele frequency (MAF) lower than 5 % were removed. Additionally, only autosomal SNPs and animals with less than 5 % of missing genotypes were retained. The final dataset for genomic-based diversity analysis included 185 individuals and 52 016 high-quality SNPs.

### 2.4 Genetic diversity assessment

After passing QC, the “–indep-pairwise” function in PLINK version 1.9 was used to prune SNPs for linkage disequilibrium (LD) by removing one of each pair of SNPs in high LD ( $r^{2}>$ 0.5) within a window size of 50 SNPs and with a window slider of 5 SNPs. Principal component analysis (PCA) was performed with PLINK to visualize the breed structure and to investigate diversity among the ram lines and *TMEM154* genotypes. The PCA plot was generated using the “ggplot2” R package (Ginestet, 2011).

Mean observed heterozygosity (Ho) and expected heterozygosity (He) were calculated for the entire breed, each ram line, and different *TMEM154* genotypes using the “hierfstat” package in R (Goudet, 2005). Ho and He were used to determine the Wright inbreeding coefficient as follows (Nei and Chesser, 1983):

1 $$F_{\mathrm{IS}}=1-\left(\frac{\mathrm{Ho}}{\mathrm{He}}\right).$$

The effective population size (Ne) was estimated for 5 (Ne_5_), 13 (Ne_13_), and 50 (Ne_50_) generations ago on the basis of LD with the “SNeP” package (Barbato et al., 2015; Weir and Hill, 1980). Additionally, Ne was calculated with published data on male (Nm) and female (Nf) population numbers (BLE, Central Documentation on Animal Genetic Resources in Germany (TGRDEU), 2024; VDL, 2023) with the following formula (Birky et al., 1983):

2 Ne=(4⋅Nm⋅Nf)/(Nm+Nf).

The expected frequencies and the number of animals per genotype class for the *TMEM154* locus were calculated using Hardy–Weinberg equilibrium (HWE) equation: $p^{2}$ + 2pq + $q^{2}$ (Mayo, 2008), where p and q represent observed frequencies of K and E alleles, respectively. Moreover, deviation of observed genotype frequencies from HWE for the *TMEM154* locus was assessed with the “HardyWeinberg” package in R using the “HWChisq()” function (Graffelman, 2015).

### 2.5 Genomic ( $F_{\mathrm{ROH}}$ ) and pedigree-based ( $F_{\mathrm{PED}}$ ) inbreeding coefficient

Prior to runs of homozygosity (ROH) analysis, QC was performed on raw genotype data of unrelated animals using the abovementioned parameters with the exception of excluding SNPs based on minor allele frequency (MAF) as proposed by Meyermans (2020b). Consequently, the dataset for the ROH analysis of the entire population included 185 individuals and 60 409 high-quality SNPs. Likewise, the number of animals per group for the ROH analysis of ram lines (nine groups) and *TMEM154* genotype (three groups) ranged from 11 to 29 and from 36 to 97, respectively. The “–homozyg” function in PLINK was used with the following settings for each run: a minimum ROH length of 1000 kb, a maximum gap between consecutive SNPs of 250 kb, at least one SNP per 100 kb, a scanning window threshold of 0.05, a sliding window of 50 SNPs, one missing SNP, and one heterozygous SNP. The minimum number of SNPs ( l ) was determined following Eq. (3) of Purfield et al. (2012):

3 $$l=\frac{\log_{e}\frac{\propto }{n_{s}n_{i}}}{\log_{e}(1-\mathrm{het})},$$

where $n_{s}$ is the number of SNPs per individual, $n_{i}$ is the number of individuals, α is the percentage of false-positive ROH results (0.05), and het is the mean SNP heterozygosity across all SNPs. The value of l was 41 for the entire RPL breed and ranged between 33 (line S) and 40 (genotype EK) in the ram lines and *TMEM154* genotypes. The total ROH number and ROH length (in Mb) were calculated for each sheep, categorized by ram line or *TMEM154* genotype, and three ROH length categories (1–5, > 5–10, and > 10 Mb) for comparison. The individual genomic inbreeding coefficient of ROH ( $F_{\mathrm{ROH}}$ ) was calculated as follows (McQuillan et al., 2008):

4 $$F_{\mathrm{ROH}}=\sum L_{\mathrm{ROH}}/L_{\mathrm{AUTO}},$$

where $\sum L_{\mathrm{ROH}}$ is the total length of all ROH segments per individual and $L_{\mathrm{AUTO}}$ is the length of the autosomal genome covered by SNPs (2.648 Gb). Box plots were constructed to visualize the within-breed distribution of $F_{\mathrm{ROH}}$ per ram line and *TMEM154* genotype for $F_{\mathrm{ROH}}$ above one Mb.

Furthermore, calculated data of pedigree-based inbreeding coefficients ( $F_{\mathrm{PED}}$ ) using serv.it OVICAP (https://www.vit.de/vit-fuers-tier/vit-fuer-schaf-und-ziege/herdbuchfuehrung-von-schafen-und-ziegen, last access: 16 August 2024), the nationwide database for breeding sheep and goats in Germany (Wilkens, 2021), provided and maintained by the company vit – “Vereinigte Informationssysteme Tierhaltung w.V.” (Verden, Germany), was retrieved from breeders. The calculation was performed by virtual mating of the parents of the analyzed individuals, which was only possible if both parents were still alive, and ewes came from the same flock. Consequently, we obtained only 27 $F_{\mathrm{PED}}$ values to compare with the $F_{\mathrm{ROH}}$ values using the Pearson rank correlation coefficient ( $r_{p}$ ) performed in SAS Studio Version 9.4 (SAS Institute Inc., Cary, NC, USA).

## 3 Results

### 3.1 *TMEM154* genotyping

Among the 191 double-genotyped samples, 181 (94.8 %) showed the same *TMEM154* genotypes as determined with the KASP technology, while the remaining 10 (5.2 %) samples had inconclusive results with KASP genotyping technology but clear results in the SNP chip analysis. Details on the distribution of *TMEM154* genotypes per ram lines and sex are given in Table 1.

After QC, the dataset for the genetic diversity analysis included 52 sheep with the genotype (frequency) KK (28 %), 97 with genotype EK (52 %), and 36 with genotype EE (20 %). The putative protective allele K was therefore predominant with 54 %.

Analysis of the expected frequencies for all genotypes revealed values of 29.2 %, 49.7 %, and 21.2 % for KK, EK, and EE, respectively. Likewise, the expected numbers of sheep for each genotype class were as follows: KK = 54, EK = 92, and EE = 39. In addition, a test for deviation from HWE was not significant ( p= 0.51) for this locus.

### 3.2 Population structure

**Figure 2:**
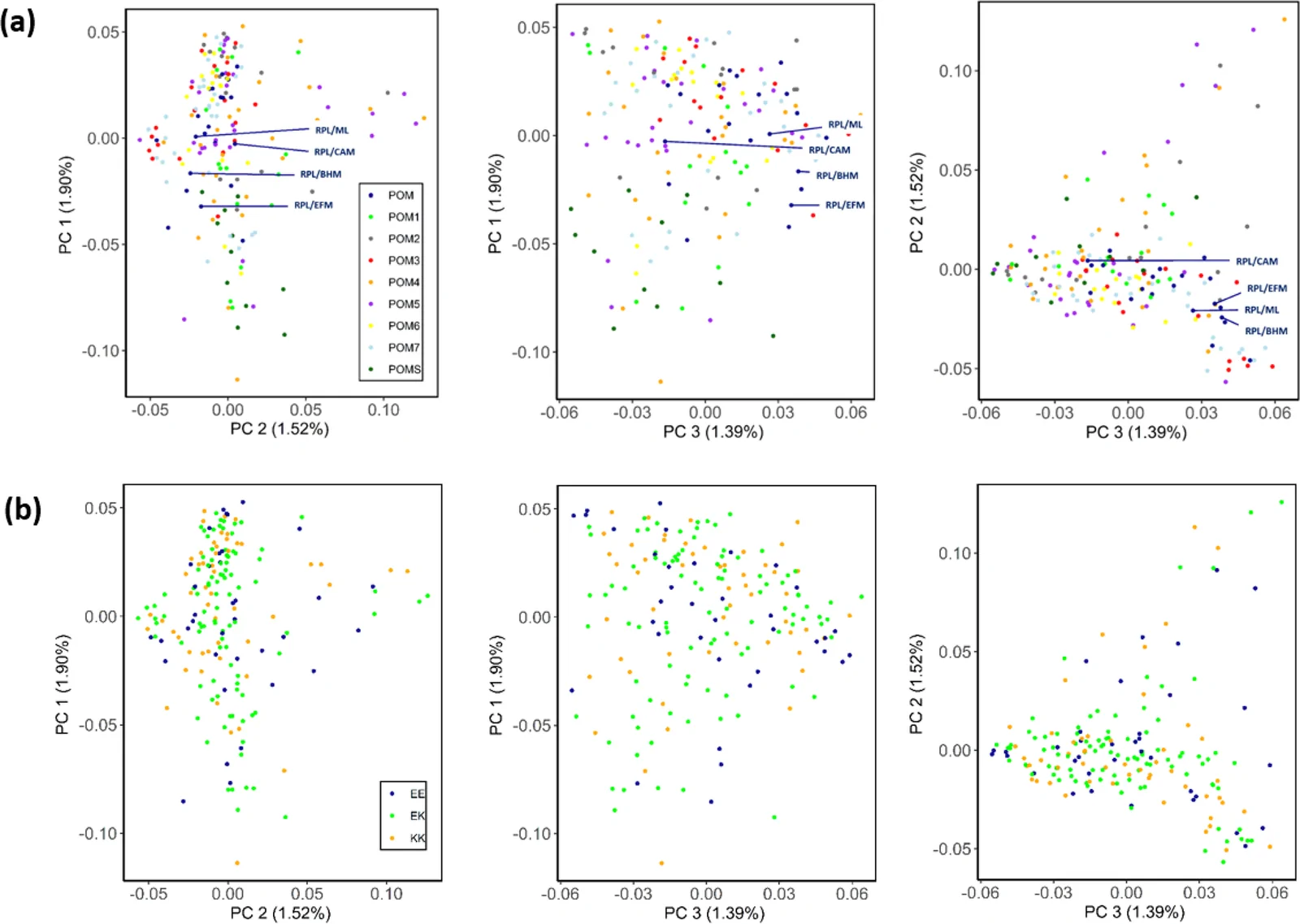
PCA plots for the first (PC1), second (PC2), and third (PC3) component of the RPL breed, categorized by ram lines **(a)** and *TMEM154* genotypes **(b)**. Abbreviations used in the figure are as follows: POM – RPL with unknown ram line; POM1–7/S – ram line 1–7/S; RPL/ML, RPL/CAM, RPL/BHM, and RPL/EFM – crossbreeds between RPL and Merino Land (ML), Cameroon (CAM), Blackheaded Mutton (BHM), and East Friesian Milk (EFM) sheep, respectively; EE, EK, and KK – *TMEM154 *genotype EE, EK, and KK at position 35, respectively.

The genetic structure of the different ram lines and *TMEM154* genotypes is illustrated in the PCA plots in Fig. 2. The first three principal components explain 4.8 % of the variation within the RPL breed. No distinct clusters were detected in any of the comparisons among the different principal components (PCs). Contrasting PC1 vs. PC3 most clearly showed the admixture of the ram lines (Fig. 2a) and the balanced distribution of the *TMEM154* genotypes (Fig. 2b).

Results of genetic diversity indices for the RPL breed, categorized by ram lines and *TMEM154* genotypes, are given in Tables 2 and 3. The diversity parameters were calculated for groups of sheep with the different *TMEM154* genotypes to determine whether subpopulations of the RPL breed with more favorable or less favorable diversity values would be favored when selecting for MVV resistance. The mean Ho, He, and $F_{\mathrm{IS}}$ values of the entire RPL breed were 0.377, 0.379, and 0.005, respectively (Table 2). Genetic diversity was very similar between the *TMEM154* genotypes, with Ho ranging from 0.374 (EK) to 0.378 (EE) (Table 2). In the ram lines, Ho ranged from 0.365 (line 2) to 0.395 (line S), while He ranged from 0.372 (line 2) to 0.385 (unknown line) (Table 3). The $F_{\mathrm{IS}}$ values were all close to zero, with three ram lines showing slightly negative results (lines 1, 3, and S).

**Table 2:** Genetic diversity (Ho, He, and $F_{\mathrm{IS}}$ ) and effective population size (Ne_5_, Ne_13_, and Ne_50_) calculated for the total breed and different *TMEM154* genotypes.

*TMEM154*	n	Ho	He	$F_{\mathrm{IS}}$	CI95 %	Ne_5_	Ne_13_	Ne_50_
E35K genotype								
EE	36	0.378	0.381	0.007	0.006–0.009	43	76	385
EK	97	0.374	0.382	0.020	0.019–0.021	66	107	596
KK	52	0.376	0.380	0.012	0.010–0.014	51	87	459
EK and KK	149	0.375	0.381	0.017	0.016–0.018	72	113	650
All RPL	185	0.377	0.379	0.005	0.003–0.008	75	116	677

**Table 3:** Genetic diversity (Ho, He, and $F_{\mathrm{IS}}$ ) calculated for the different ram lines.

Ram line	*n*	Ho	He	$F_{\mathrm{IS}}$	CI95 %
Line 1	15	0.381	0.380	- 0.001	- 0.004–0.001
Line 2	17	0.365	0.372	0.019	0.017–0.022
Line 3	19	0.380	0.378	- 0.004	- 0.006 to - 0.002
Line 4	28	0.373	0.379	0.015	0.014–0.018
Line 5	28	0.373	0.377	0.011	0.009–0.013
Line 6	17	0.374	0.375	0.002	- 0.000–0.004
Line 7	29	0.374	0.379	0.013	0.010–0.015
Line S	11	0.395	0.382	- 0.035	- 0.039 to - 0.032
Unknown line	21	0.375	0.385	0.028	0.026–0.030

Effective population size (Ne) was estimated using the QC dataset for the entire breed and for groups of the different *TMEM154* genotypes for 5, 13, and 50 generations ago (Table 2). The Ne for all samples and the combined genotypes KK and EK were the highest, with Ne_5_ values of 75 and 72, respectively. The Pearson correlation coefficient ( $r_{p}$ ) between these two Ne_5_ values was almost 1 ( $r_{p}=$ 0.99, p<0.0001 ). The homozygous genotypes EE and KK had lower Ne values, which can be explained by their limited sample size.

**Figure 3:**
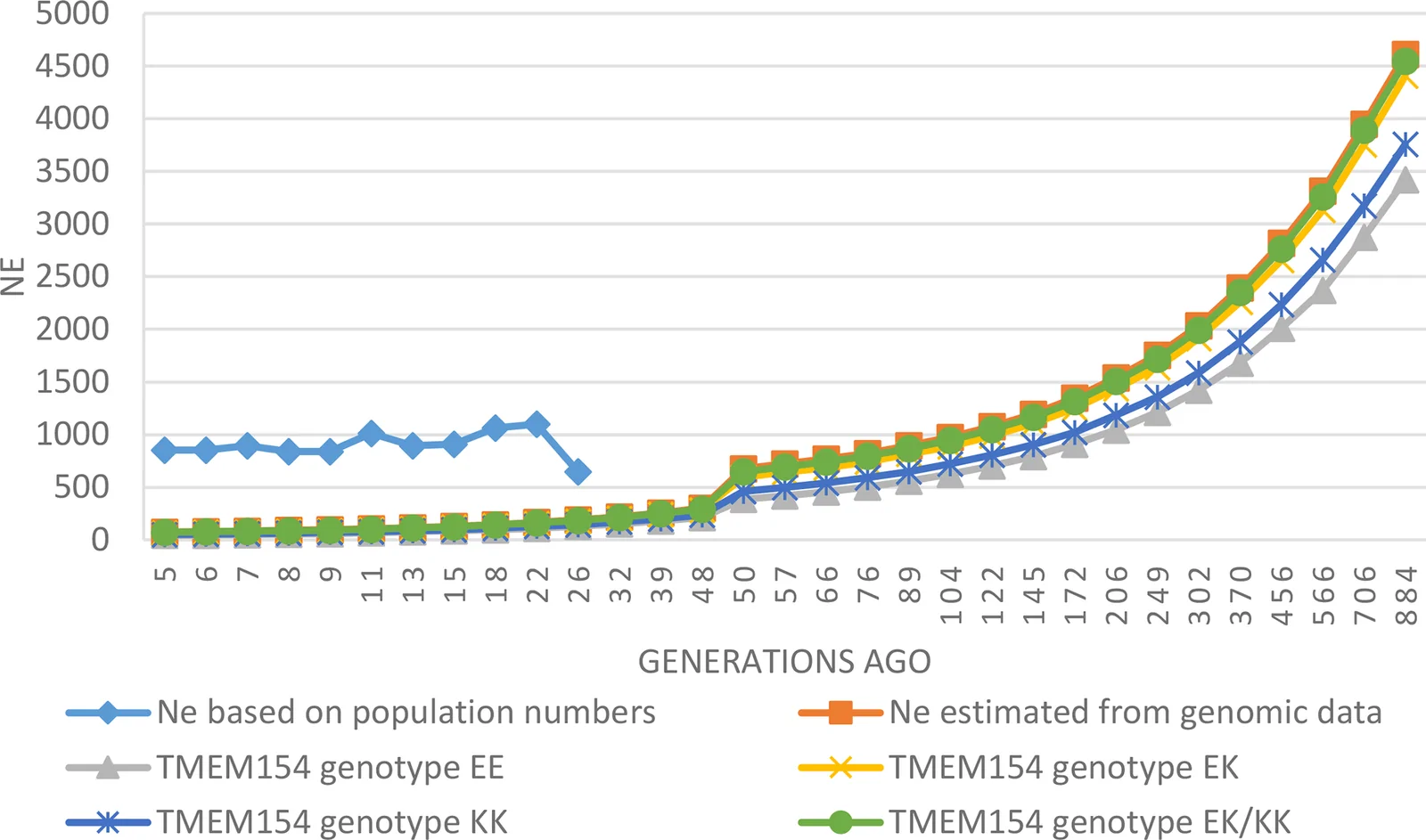
Ne based on population numbers across 26 generations and estimated genomic Ne across 884 generations for the total breed and the different *TMEM154* genotypes.

Ne based on the numbers of male and female RPL animals showed substantial differences compared to the genomic Ne ( $r_{p}=$ 0.03, p= 0.93). This is illustrated in Fig. 3, which shows these differences and the development of Ne over the past 800 generations. All genomic Ne values show a declining trend, while Ne values based on population numbers have mostly increased in recent generations, with Ne_5_ and Ne_13_ reaching 852 and 896, respectively. For the year 2023, Ne was estimated to be 1013.

### 3.3 Runs of homozygosity

A total of 6690 ROH segments with varying lengths ranging between 1.06 and 38.24 Mb were identified for *TMEM154* genotypes. With one exception, all animals had at least 4 and a maximum of 116 ROH segments (Appendix A). On average, this resulted in 16 ROH segments per individual within the entire RPL breed.

**Figure 4:**
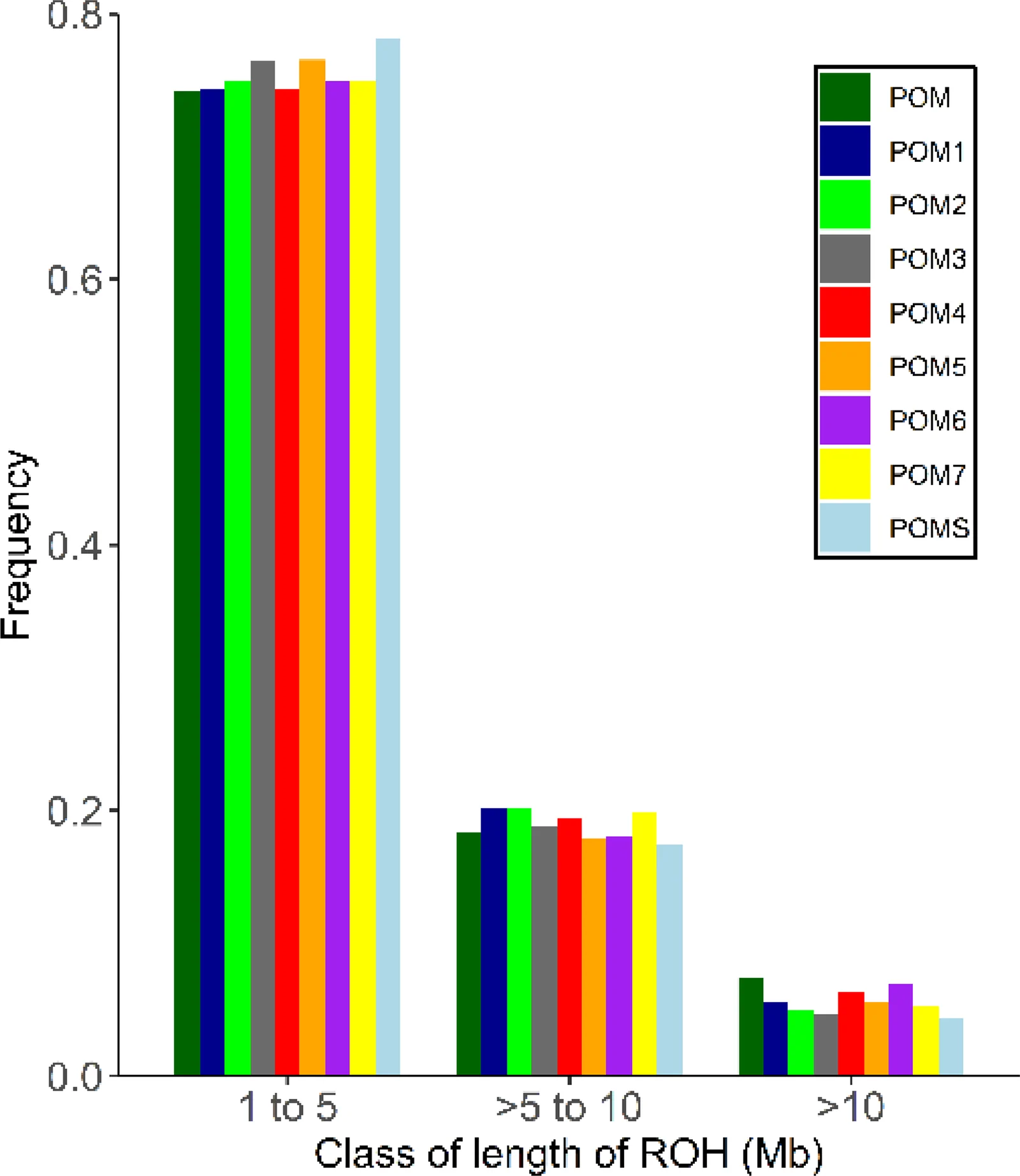
Frequency distribution of the number of ROH in different length classes and for the RPL breed, categorized by ram lines. Abbreviations used in the figure are as follows: POM – RPL with unknown ram line; POM1–7/S – ram line 1–7/S.

When comparing the different RPL ram lines, animals with unknown line assignment had the highest number of ROH segments, while line 4 had the longest ROH (Appendix B). The majority of ROH segments were in the shortest length class (1–5 Mb), with frequencies between 74.15 % (unknown line) and 78.18 % (line S), followed by the intermediate class ( > 5–10 Mb), ranging from 17.46 % (line S) to 20.17 % (line 1). Only 4.37 % (line S) to 7.45 % (unknown line) of the ROH segments were longer than 10 Mb (Fig. 4). The distributions of the frequencies of ROH segments in different length classes (1–5, > 5–10, and > 10 Mb) and the ram lines are shown in Fig. 4.

**Figure 5:**
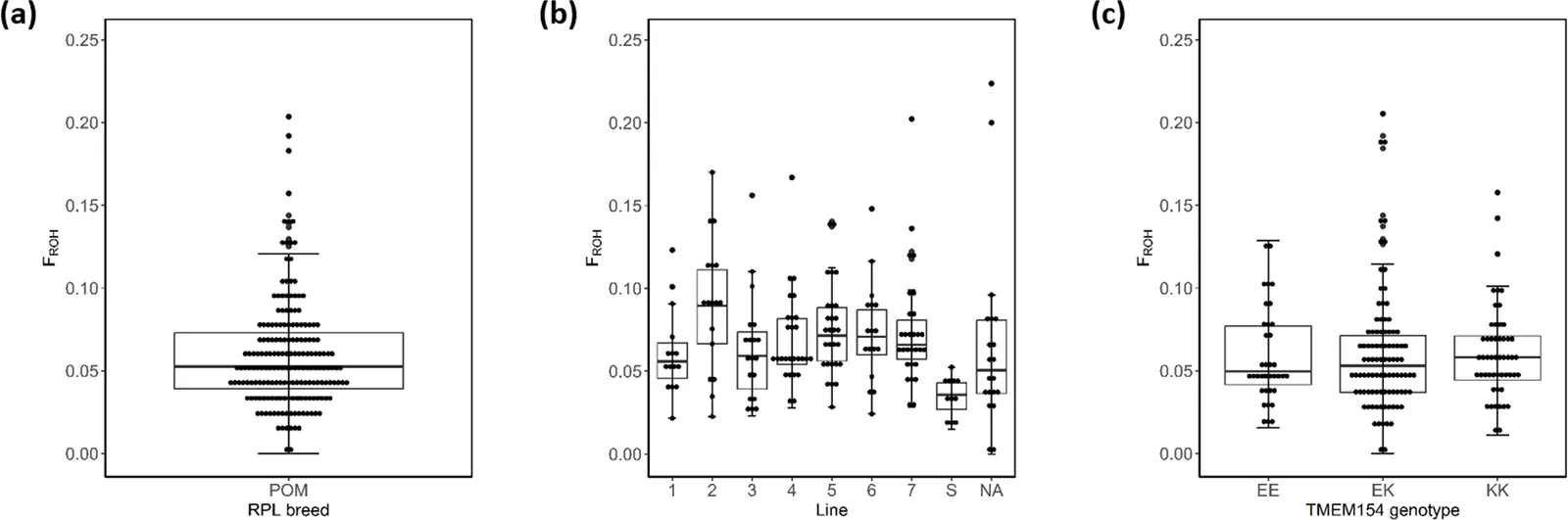
Box plots of within-breed $F_{\mathrm{ROH}}$ for the entire RPL breed **(a)**, the ram lines **(b)**, and *TMEM154* genotypes **(c)**. Abbreviations used in the figure are as follows: POM – all 185 RPL; 1–7 – ram line 1–7; NA – line data not available; EE, EK, and KK – *TMEM154* genotype EE, EK, and KK at position 35, respectively.

Based on ROH analysis, genomic inbreeding ( $F_{\mathrm{ROH}}$ ) was estimated for the entire breed, for *TMEM154* genotypes (Appendix A), and separately for the different ram lines (Appendix B). In the RPL breed, $F_{\mathrm{ROH}}$ values ranged from 0 to 0.277, with a mean of 0.062. The within-breed distribution of $F_{\mathrm{ROH}}$ is visualized in Fig. 5, where the bottom of the box represents the third quartile (Q3), followed by the median and the first quartile (Q1).

The highest mean $F_{\mathrm{ROH}}$ was found in genotype EK (0.065), while genotype EE had the lowest (0.058) (Appendix A). Otherwise, the highest median was presented in genotype KK (Fig. 5c). The Kruskal–Wallis test indicated no significant differences between the mean $F_{\mathrm{ROH}}$ of the *TMEM154* genotypes ( p= 0.836).

A significant difference was observed between the $F_{\mathrm{ROH}}$ of the eight ram lines ( p= 0.0002) (Appendix C). Only the $F_{\mathrm{ROH}}$ comparisons between line S and six other lines, with the exception of line 3, were significant. The highest median and mean value were observed in line 2, whereas the lowest values were observed in line S (Appendix B, Fig. 5b).

In 27 individuals for which a pedigree-based inbreeding coefficient ( $F_{\mathrm{PED}}$ ) could be calculated, the $F_{\mathrm{ROH}}$ and $F_{\mathrm{PED}}$ values were compared (Appendix D). The $F_{\mathrm{ROH}}$ values of these animals ranged between 1.12 % and 12.90 % (average of 5.46 %), whereas the $F_{\mathrm{PED}}$ values were consistently lower, ranging from 0 % to 1.56 % (average of 0.44 %). A value of 0 % indicates no inbreeding, either genomic ( $F_{\mathrm{ROH}}$ ) or pedigree-based ( $F_{\mathrm{PED}}$ ). The Pearson correlation coefficient ( $r_{p}$ ) between $F_{\mathrm{ROH}}$ and $F_{\mathrm{PED}}$ was positive and moderately significant, with a result of 0.42 ( p= 0.02).

## 4 Discussion

### 4.1 Genetic diversity in the RPL breed

This study presents the first comprehensive analysis of genetic diversity in the German RPL sheep breed. Faced with the danger of extinction, efforts to conserve the breed began almost 40 years ago with seven rams from the eastern federal state Mecklenburg–Western Pomerania and one ram from southwestern Germany (IGRPL, 2024). Today, these eight founding ram lines are distributed across the whole country (VDL, 2023). The PCA plot (Fig. 2a) indicates an equal genetic distribution with no distinct clustering among the ram lines. A genetic separation of individuals from different geographical regions in PCA plots, as reported in previous studies of different breeds (Eydivandi et al., 2021; Kijas et al., 2009; Kijas et al., 2012), could not be observed in the present study. Line S, originating from southern Germany, showed no distinct separation from the seven northern lines, despite its different geographical origin. This outcome speaks for a successful implementation of the line-rotation method by the RPL breeders. Normally, the genetic distance among animals from the same breed is smaller than the distance between animals of divergent breeds (Lenstra et al., 2012). However, the four crossbred RPL sheep (resulting from crosses with Cameroon, Blackheaded Mutton, Merino Land, and East Friesian Milk sheep) exhibited no clearly visible genetic distance from the purebred individuals in the PCA plot. For identification of crossbred animals with this method, it may be necessary to include SNP data from sheep of the other breeds. In fact, the genetic proportion of the crossed-in breed also depends on how many generations ago the cross was made. Unfortunately, we do not have this information for the crossbred animals included in this study.

Numbers of breeding males and females have traditionally been used to estimate Ne in livestock (Birky et al., 1983). However, these estimates have limitations, such as assumptions of perfect random mating, including self-fertilization, or an equal number of offspring per parent. Ne based on genomic data serves as a more accurate indicator of the genetic variation within a population and is usually much smaller than Ne based on population numbers (Lenstra et al., 2012). When using the SNeP software for Ne analysis, the authors caution that Ne values based on genome data are unreliable when calculated for less than 10 generations (Corbin et al., 2012). Therefore, genome-based Ne values lower than 10 generations ago found in this study are not comparable to results from population numbers.

The present study supports the latter finding, e.g., with Ne_13_ values of 116 (genomic) and 896 (population numbers) for all analyzed samples. Both values are over the threshold recommended guideline value of at least 50 from the Food and Agriculture Organization of the United Nations (Bradley et al., 1998), indicating that the RPL breed is not currently at risk of extinction or inbreeding depression. The genomic Ne of the RPL breed is higher than those reported for many other sheep breeds (Adeniyi et al., 2022; Meyermans et al., 2020a; Purfield et al., 2017). Comparable or even higher values have been reported in some Swiss sheep breeds (Signer-Hasler et al., 2019). Overall, the genomic Ne of the RPL breed shows a decreasing trend over the last 800 generations (Fig. 3). This decline aligns with population subdivision and selection (Kijas et al., 2012), and such a decline is to be expected for breeding in a closed population.

The still acceptable effective population size of the RPL breed is consistent with the results for other parameters of genetic diversity. A higher level of heterozygosity indicates greater genetic variability, while a lower level suggests both lower genetic variability and a lower Ne (Lenstra et al., 2012). The mean observed heterozygosity (Ho) of the RPL breed is higher than that of 10 northern European sheep breeds (Meyermans et al., 2020a) and nearly matches the Ho value of 0.38 in Australian Merino sheep, which have been described as the most diverse sheep population among a selection of Australian breeds (Al-Mamun et al., 2015). Other European sheep breeds exhibited both lower and higher values (of up to 0.41) (Adeniyi et al., 2022; Signer-Hasler et al., 2019; Sveistiene and Tapio, 2021). Within RPL sheep, the S-line had the highest heterozygosity (0.40), comparable to that of the German Texel breed (Sveistiene and Tapio, 2021). A German diversity study from 1999 using microsatellites estimated a heterozygosity of 0.77 for the RPL breed (Falge et al., 1999). The phenomenon that microsatellite data lead to higher estimates of heterozygosity than SNP chip genotypes has already been described (Handley et al., 2007). Microsatellite analyses show higher heterozygosity because most of these markers represent highly polymorphic sites with more than two alleles, in contrast to SNPs that usually represent sites with two alleles. However, SNP chip genotypes are more accurate than microsatellites due to the higher number of markers and their more even distribution across the genome. Therefore, they are able to detect even weak relationships, and they result in smaller confidence intervals (Laoun et al., 2020). The inbreeding coefficient $F_{\mathrm{IS}}$ showed both positive and negative results across the different ram lines, although all were close to 0. Other German breeds, such as Blackheaded Mutton, East Friesian Brown, or Texel sheep, showed negative results ranging from - 0.02 to - 0.03 based on SNP chip data (Sveistiene and Tapio, 2021). The mean $F_{\mathrm{IS}}$ value for the RPL breed was slightly positive, which indicates a slight tendency towards inbreeding.

The majority of the ROH segments across all ram lines belonged to the shortest length class (Fig. 4), which indicates older inbreeding events, such as bottlenecks during breed formation due to the small initial population (Curik et al., 2014; Peripolli et al., 2017). Only a small proportion of long ROH segments were detected, suggesting low recent inbreeding (Curik et al., 2014). This recent inbreeding could be attributed to selective breeding and non-random mating, as usually only a small number of rams are used. The genomic inbreeding coefficient $F_{\mathrm{ROH}}$ aligns with the $F_{\mathrm{IS}}$ values, with the highest $F_{\mathrm{ROH}}$ average found in line 2 (0.090) and the lowest in line S (0.035) (Appendix A). The overall breed value was 0.062, which is far below the average of 0.134 for European breeds (Eydivandi et al., 2021) and other endangered sheep breeds (Adeniyi et al., 2022; Meyermans et al., 2020a).

When comparing the genomic inbreeding coefficient ( $F_{\mathrm{ROH}}$ ) with the pedigree-based inbreeding coefficient ( $F_{\mathrm{PED}}$ ), the $F_{\mathrm{ROH}}$ values were consistently higher than the $F_{\mathrm{PED}}$ values, with a correlation of only 0.42. Moderate to weak correlations were also detected in the Vendéen breed (value of 0.15) (Purfield et al., 2017) and some Swiss sheep and goat breeds (Burren et al., 2016; Signer-Hasler et al., 2019). In contrast, other studies on sheep, pigs, and cattle have reported higher correlations (Gorssen et al., 2020; Meyermans et al., 2020a; Signer-Hasler et al., 2017), while dogs showed the highest at 0.78 (Wiener et al., 2017). The discrepancy could be explained by a higher degree of incompleteness and lower depth of pedigrees of small ruminants (Purfield et al., 2017). In comparison, pets generally have a better-documented pedigree due to easier control of mating and birth. Regarding birth, sheep and goats often lamb in groups and at night. An overlooked adoption of foreign lambs by ewes can lead to errors in the pedigree. Control of mating is also an advantage of artificial insemination, which (at least in Europe) is more common in cattle and pigs than in sheep and goats. Moreover, $F_{\mathrm{PED}}$ is based on theoretical inbreeding and does not consider the stochastic effect of Mendelian sampling (Curik et al., 2014). Another possible explanation for lower correlations is the assumption that the founder population is unrelated, which is unlikely. Due to these limitations, $F_{\mathrm{PED}}$ often underestimates inbreeding. Consequently, $F_{\mathrm{ROH}}$ is more accurate because it is independent of ancestry information and reflects both past and recent relatedness (Curik et al., 2014; Peripolli et al., 2017).

### 4.2 Breeding for maedi-visna resistance

Based on the diversity parameters calculated in the present study, the genetic diversity of the RPL breed is not at risk. Due to the high frequency of the *TMEM154* allele K in this study (54 %) and a previous study (53 %) (Frölich et al., 2024), breeding for MVV resistance could be possible. The PCA results showed no genetic separation between sheep with the three different *TMEM154* genotypes. This implies that no genetically distinct subgroup will be excluded from breeding when selecting for the K allele. However, a strong preference in breeding for the putative protective allele K might jeopardize the genetic variability in this breed and increase inbreeding rates by reducing the effective population size (Peripolli et al., 2017; Williams, 2005). The estimated Ne for the subpopulation of sheep with the *TMEM154* genotype KK was slightly lower than that of the entire breed, whereas the Ne for sheep with genotypes KK and EK was similar (Fig. 3). Implementing mating strategies that include both genotypes (KK and EK) would not substantially reduce Ne and, consequently, help maintain allelic variation (Peripolli et al., 2017). Moreover, the observed heterozygosity (Ho) remained the same, whereas the expected heterozygosity (He) was higher for *TMEM154* genotypes KK and EK (Table 2), resulting in a slightly higher but still near-zero $F_{\mathrm{IS}}$ value. On the other hand, the mean $F_{\mathrm{ROH}}$ value of 0.063 for both genotypes was comparable to that of the whole breed. Although the *TMEM154* genotype EE exhibited the lowest $F_{\mathrm{IS}}$ and $F_{\mathrm{ROH}}$ results, a selection against this genotype is not problematic due to its reactively low frequency in the RPL population (20 %). To reduce genotyping costs, sire-only genotyping and selection should be considered. While this approach would slow the selection for the K allele, it would avoid high costs and limit the reduction in the population size (White and Knowles, 2013).

However, the effectiveness of selection for the *TMEM154* allele K with respect to reducing MVV susceptibility in the RPL breed needs further validation through studies involving a larger number of MVV-positive sheep, as the effect showed only a trend towards significance (Frölich et al., 2024). Nonetheless, it has the potential to complement MVV eradication measures in infected flocks, such as regular serological monitoring of the flock and culling of seropositive sheep and/or motherless rearing of lambs (Reina et al., 2009). In the light of the high frequency of the K allele in the RPL breed, a prioritization of breeding rams with the genotypes KK or EK in MVV-infected flock is a relatively simple way to increase the chances of reducing the infection pressure within the flock.

## 5 Conclusion

This study describes, for the first time, the genomic diversity of the German RPL sheep breed. The estimated genetic diversity parameters suggest that its diversity is not at risk, indicating successful breeding practices in recent years. Nevertheless, it is important to maintain genetic diversity and further reduce the level of inbreeding in the future. For this purpose, the genomic inbreeding coefficient should be used, as the pedigree-based method consistently underestimated the inbreeding level. However, to enable this, it would be necessary to establish genome analysis as a routine procedure in German sheep breeding.

Furthermore, the present study showed that breeding for MVV resistance would be possible without a major loss of genetic diversity. However, selecting the entire breed for the *TMEM154* allele K is not recommended at this time, as the effect on MVV susceptibility was not statistically significant in a previous study. An exception are MVV-affected flocks, in which rams with the genotypes KK or EK could be prioritized. Further research with a larger number of MVV-positive RPL sheep is required in order to validate a protective effect of the genotype KK against MVV in the RPL breed.

## Data Availability

As the data originate from German sheep farms, there are privacy issues associated with their public release. Therefore, the data are only available from the authors upon reasonable request, as permission must first be obtained from the sheep farmers.

## Appendix A

**Table A1:** Descriptive statistics for the ROH analysis and $F_{\mathrm{ROH}}$ of the total breed and the *TMEM154* genotypes.

*TMEM154*		ROH length (Mb)		ROH number		$F_{\mathrm{ROH}}$	
E35K genotype	n	Mean (SD)	Range	Mean (SD)	Range	Mean	Range
EE	36	4.28 (2.78)	1.06–33.77	36.17 (15.29)	12–69	0.058	0.015–0.129
EK	97	4.55 (3.14)	1.27–38.24	37.52 (20.24)	0–118	0.065	0–0.261
KK	52	4.38 (2.74)	1.10–25.70	36.77 (14.65)	9–79	0.061	0.011–0.158
EK and KK	149	4.52 (3.01)	1.10–38.24	36.92 (18.28)	0–118	0.063	0–0.261
Total	185	4.52 (2.98)	1.06–38.24	36.16 (17.50)	0–116	0.062	0–0.259

## Appendix B

**Table B1:** Descriptive statistics for the ROH analysis and $F_{\mathrm{ROH}}$ of the eight different ram lines.

		ROH length (Mb)		ROH number		$F_{\mathrm{ROH}}$	
Ram line	n	Mean (SD)	Range	Mean (SD)	Range	Mean	Range
Line 1	15	4.21 (3.24)	1.05–36.16	38.67 (14.12)	15–67	0.061	0.022–0.123
Line 2	17	4.25 (3.09)	1.01–38.39	56.12 (20.46)	15–86	0.090	0.023–0.170
Line 3	19	4.09 (2.87)	1.07–25.37	41.47 (15.59)	19–82	0.064	0.023–0.156
Line 4	28	4.42 (3.80)	1.10–64.46	46.14 (19.16)	20–108	0.077	0.028–0.280
Line 5	28	4.12 (2.83)	1.02–28.21	48.54 (14.21)	19–81	0.075	0.028–0.140
Line 6	17	4.35 (3.42)	1.02–35.70	44.59 (14.50)	17–79	0.073	0.024–0.148
Line 7	29	4.25 (3.17)	1.05–36.25	46.83 (15.32)	20–88	0.075	0.028–0.202
Line S	11	3.99 (2.75)	1.07–21.24	22.91 (8.14)	11–34	0.035	0.015–0.052
Unknown line	21	4.60 (3.89)	1.08–31.04	43.48 (29.51)	0–121	0.076	0–0.272

## Appendix C

**Table C1:** Results of the Kruskal–Wallis test comparing the eight ram lines.

Line comparison	p value
6 vs. 5	1.000
6 vs. 4	1.000
6 vs. 2	0.902
6 vs. 3	0.959
6 vs. 1	0.924
6 vs. 7	1.000
6 vs. S	0.012
5 vs. 4	0.997
5 vs. 2	0.755
5 vs. 3	0.822
5 vs. 1	0.666
5 vs. 7	1.000
5 vs. S	0.001
4 vs. 2	0.769
4 vs. 3	0.975
4 vs. 1	0.940
4 vs. 7	0.999
4 vs. S	0.001
2 vs. 3	0.390
2 vs. 1	0.393
2 vs. 7	0.775
2 vs. S	0.009
3 vs. 1	1.000
3 vs. 7	0.956
3 vs. S	0.154
1 vs. 7	0.835
1 vs. S	0.036
7 vs. S	0.001

## Appendix D

**Table D1:** Comparison of the genomic ( $F_{\mathrm{ROH}}$ ) and pedigree-based ( $F_{\mathrm{PED}}$ ) inbreeding coefficients for 27 pedigreed RPL individuals.

Pedigreed individual	$F_{\mathrm{ROH}}$ (%)	$F_{\mathrm{PED}}$ (%)
1	4.04	0.00
2	6.37	1.56
3	2.41	0.00
4	3.56	0.00
5	5.38	0.78
6	2.74	0.00
7	7.20	1.17
8	3.57	0.98
9	3.19	0.39
10	6.07	0.00
11	7.60	0.98
12	10.12	0.20
13	6.99	0.39
14	2.01	0.00
15	8.19	1.56
16	5.33	0.00
17	4.80	0.98
18	6.87	0.00
19	4.40	0.00
20	8.62	0.39
21	12.90	0.52
22	8.87	1.17
23	4.33	0.78
24	1.95	0.00
25	1.12	0.00
26	4.16	0.00
27	4.68	0.00

## Code availability

The statistical codes used in this study are available from the corresponding author upon reasonable request.
